# Cleaning Staff’s Attitudes about Hand Hygiene in a Metropolitan Hospital in Australia: A Qualitative Study

**DOI:** 10.3390/ijerph16061067

**Published:** 2019-03-25

**Authors:** Marguerite C. Sendall, Laura K. McCosker, Kate Halton

**Affiliations:** 1 School of Public Health and Social Work, Queensland University of Technology, Victoria Park Road, Kelvin Grove QLD 4159, Australia; lkmccosker@ozemail.com.au; 2 Institute of Health and Biomedical Innovation, Queensland University of Technology, Musk Avenue, Kelvin Grove QLD 4159, Australia; k.halton@gmail.com

**Keywords:** hand hygiene, hospital cleaners, infection control, qualitative research, attitudes

## Abstract

Background: In 2009, the National Hand Hygiene Initiative (NHHI) was implemented in hospitals across Australia with the aim of improving hand hygiene practices and reducing healthcare-associated infections. Audits conducted post-implementation showed the lowest rates of compliance with hand hygiene practices are among operational staff including hospital cleaners. There is limited information about hand hygiene issues in hospital cleaners to inform development of evidence-based interventions to improve hand hygiene compliance in this group. Aim: This qualitative study was undertaken to explore the attitudes of hospital cleaning staff regarding hand hygiene and the National Hand Hygiene Initiative. Methodology: Focus groups were conducted with 12 cleaning staff at a large Australian hospital implementing the National Hand Hygiene Initiative. Findings: Hospital cleaners recognise the importance of hand hygiene in preventing healthcare-associated infections. Cleaners cite peer support, leadership, and the recognition and reward of those excelling in hand hygiene as strong motivators. Barriers to optimal hand hygiene practice include the presence of multiple conflicting guidelines, hand hygiene “overload” and a lack of contextualised education programs. This exploratory qualitative study reveals three themes about attitudes of hospital cleaning staff towards hand hygiene. These themes are: (1) “The culture of hand hygiene: It’s drummed into us”; (2) “Reminders and promotion for hand hygiene: We just need a big ‘Please wash your hands’ sign”; and (3) “The personal value of hand hygiene: Like he said, it’s second nature to us”. Conclusion: Hand-hygiene messages and training need to be more consistent and contextualised to achieve improvements in hand hygiene practices in hospital cleaning staff in Australia.

## 1. Introduction

A cornerstone of good hospital infection control is adequate hand hygiene practices in all staff who have contact with patients or the patient environment [1,2,3]. In 2009, the National Hand Hygiene Initiative (NHHI) was implemented in hospitals across Australia with the aim of improving hand hygiene practices and reducing health care associated infections [4,5]. In the hospital where this study was carried out, a substantial decline in infection rates was achieved in *Staphylococcus aureus* infections. From 2013–2014 to 2016–2017, there was a decline from a high of 72 cases to 34 cases [6].

Audit results however show the lowest rates of hand hygiene compliance continue to be amongst non-clinical staff: 65% amongst hospital cleaning staff compared with 81% amongst nurses and midwives [7]. Whilst hospital cleaning staff are not involved in direct patient care, they have frequent contact with the patient environment and this could lead to the transfer of infectious organisms. Most existing research has focused on hand hygiene practice amongst staff in clinical roles [2,3]. There is limited information about hand hygiene in cleaning staff to inform development of evidence-based interventions to improve compliance in hospital cleaning staff. As such, the objective of this qualitative case study was to identify attitudes toward hand hygiene and the NHHI amongst hospital cleaners.

**Diagram 1:**
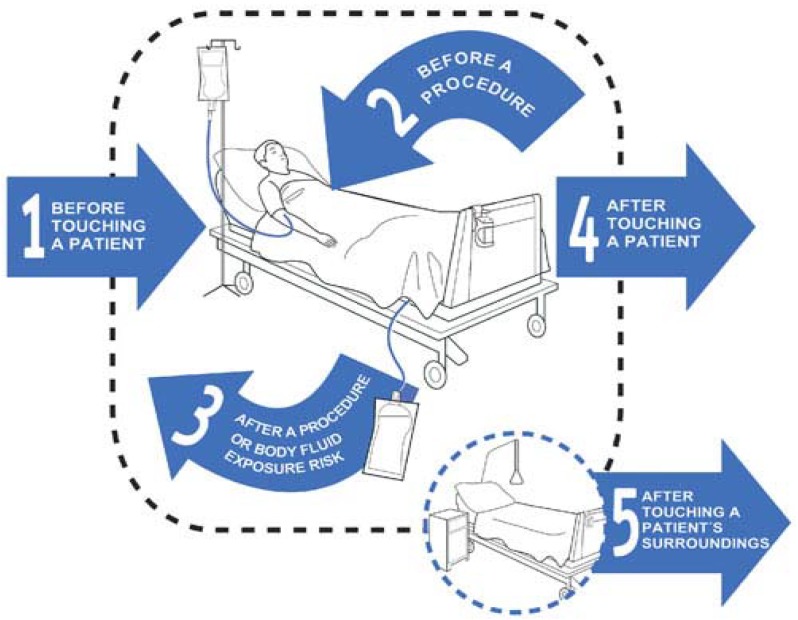
Five Moments for Hand Hygiene [5].

In 2009, the Five Moments for Hand Hygiene program was implemented in hospitals across Australia under the National Hand Hygiene Initiative (NHHI) [5]. Five moments in time have been identified as critical when hand hygiene is performed. The Five Moments for Hand Hygiene program relates to workers cleaning their hands after touching the patient’s immediate surroundings, including inanimate surface and objects when the patient has not been touched. The program is designed for operational officers, including environmental services officers, ward personnel, catering staff, linen services and support officers, as well as all healthcare workers in clinical roles. Operational staff are not involved in direct patient care and are less likely to inoculate patients. However, they can be responsible for contaminating patients’ environment, which can lead to the transfer of pathogens. Refer to Diagram 1: Five Moments for Hand Hygiene. 

In 2009, Queensland’s Strategic Operational Services Advisory Network (SOSAN) raised a number of concerns about the performance of the Five Moments for Hand Hygiene program among operational staff and what this meant for practice and work processes in this professional group. Since the commencement of the NHHI, operational staff have shown low compliance rates to hand hygiene practices. Between January 2008 and December 2009, the compliance rate of operational staff with the Five Moments for Hand Hygiene program was 44% compared with 51% amongst all healthcare workers.

## 2. Methodology

This study was conducted in a large hospital in Brisbane, Australia. A pragmatic qualitative study design [8] using focus group discussions was conducted to elicit information from cleaning staff about their attitudes to hand hygiene and the NHHI. 

*Recruitment:* Hospital cleaning staff were recruited, with their supervisors’ support, via posters pinned on relevant notice boards and flyers left in staff common areas. Support was provided to backfill staff, allowing cleaning staff to participate in focus groups held onsite during work hours. In total, 12 hospital cleaners participated in two focus groups: eight in the first and four in the second.

*Data collection:* Data were collected between January and March 2013 by two female researchers. Participants were given time to read a participant information sheet, which included information about measures to ensure confidentiality, the voluntary nature of the research, and potential risks and benefits from participation. Each participant signed a consent form, including permission for audio recording. Focus groups took 60 min and were directed by a guide consisting of 10 broad, open-ended questions. Refer to Appendix A: Focus Group Discussion Guide. All research was carried out in the hospital staff room to provide a familiar setting. Probing questions were used to explore responses in more detail to elicit rich, textured data. At the conclusion of each focus group, the researchers collected demographic data about the participants’ gender, age and years of employment (Table 1). 

*Data analysis:* Data were analysed thematically using open and axial coding. Audio recordings were transcribed by a transcription service. One researcher de-identified transcripts, assigning participant details with generic codes for data analysis, storage and retrieval. Two researchers listened to each audio-recording to gain a sense of the “wholeness” of the data, and identified significant statements in context and key concepts in each transcription. A code was attached to each statement/concept to represent its underlying premise and then codes were grouped to identify common ideas within each of the concepts explored. An iterative and cylindrical process of re-coding and re-grouping ideas was undertaken by hand until such time the researchers felt the data had “settled” into themes. A satisfactory co-judge concordance of over 90% was achieved to demonstrate rigour was adhered to throughout the analysis [9]. Mismatches were discussed, and researchers agreed to agree or disagree. Throughout the process, internal and external validity were maintained by not rushing to finalise analysis. Finally, each theme was given a descriptive label.

Ethics approval for this project was granted by the Queensland Health Human Research Ethics Committee (approval number: HREC/12/QRCH/203) and the Queensland University of Technology Human Research Ethics Committee (approval number: 1200000714). All participants provided written informed consent for participation. 

## 3. Results

Three main themes related to hospital cleaning staff’ hand hygiene-related attitudes were identified: (1) “The culture of hand hygiene: “It’s drummed into us”; (2) “Reminders and promotion for hand hygiene: We just need a big ‘Please wash your hands’ sign”; and (3) “The personal value of hand hygiene: Like he said, it’s second nature to us”. 

Theme 1: The culture of hand hygiene: “It’s drummed into us”. 

The first theme is labelled “The culture of hand hygiene: It’s drummed into us”. This theme encompasses three salient perceptions about the culture of hand hygiene: the influence of peers and reward and recognition amongst peers, supervisors, and hospital culture, including auditing, on hand hygiene practice. Hospital cleaning staff consider peers and supervisors have contributed to widespread change in the culture of hand hygiene and convey a sense of perverseness across the hospital environment. 

Participants perceived peers to be influential in supporting and promoting good hand hygiene practice. In the hospital, staff in clinical areas with excellent rates of hand hygiene are awarded “life saver” shirts. This perception relates to these colourful t-shirts obvious to hospital visitors. Participants perceive these t-shirts to be popular and everyone would like one.

FGD1: A lot of the nurses wear “clean hands, lifesaver” shirt, which is a big print on the back of their shirts. “Clean hands, save lives” and a big lifesaver on the back. That is huge on the back of their shirt. I think everyone’s gone through a bit of a trend trying to get one.

Participants feel peer influence comes from a sense of competitiveness generated by reward and recognition for excellence in hand hygiene compliance. Reward and recognition is seen as a strong incentive, influential on behaviour and increases the positive culture of hand hygiene. In particular, participants reveal the t-shirts are highly prized as the reward for receiving the hand hygiene award.

FGD1: “We got one, yeah, because we got the Turner award of hand cleaning. And we got it twice now. They got a big box of T-shirts, all different sizes, random. So at the nurse’s station, we went and helped ourselves and took them, took one each. They’re good”.

Supervisors are perceived as significantly influential in supporting good hand hygiene practice. Participants indicated supportive supervisors contributed to their compliance, among peers and throughout the hospital.

FGD1: “If I went to the supervisor and said, “Look, I saw one of us walking to an isolation unit without the correct procedure…. Yeah, our supervisor would support that 100 per cent.

Participants indicate hand hygiene culture at the hospital to be a significant factor in influencing their compliance. Participants identify that a change in hand hygiene culture had occurred over the past ten to fifteen years; now, hand hygiene is strongly emphasised within the hospital and cleaning staff undertake hand hygiene education every six months. Hand hygiene is perceived to be everybody’s responsibility”.

FGD1: “It’s drummed into us over a period that it’s the right thing to do, to benefit everybody; not just yourself, the whole hospital, the patients, the other staff, everybody. We all do it”.

Participant reveal an aspect of hand hygiene culture is auditing with a focus on feedback and monitoring. Receiving audit performance feedback has been particularly useful in making hospital cleaning staff more mindful of hand hygiene: Hospital cleaning staff are aware of ward audits but are not intimidated by them because they feel confident in their hand hygiene practice:

FGD2: “It is second nature to us to wash our hands, it doesn’t matter if someone is watching or not, we do it anyway out of respect to patients”.

Theme 2: “Reminders and promotion for hand hygiene: We just need a big ‘Please wash your hands’ sign”.

The second theme is titled “Reminders and promotion for hand hygiene: We just need a big ‘Please wash your hands’ sign”. This theme encapsulates three germane perceptions: confusion from an overload of different, generic, non-tailored hand hygiene information sessions, online training modules, posters and reminders, becoming accustomed to messages and proposes the notion of simple reminders and complimentary activities.

Participants perceived many hand hygiene guidelines are currently promoted which, despite the apparent simplicity of the Five Moments-based programme, created confusion about the exact practices necessary for optimal hand hygiene—for example, when exactly to change gloves within the ward:

FGD2: “With clinical I am pretty anal when it comes to wearing gloves. I whack them on. Just to basically protect myself and any suspicion that the outside world could be looking at me, when I am not wearing them, I’m not sure when the exact time to put them on is, but I do it just in case”.

Most participants agree there is a “poster overload”, that is, there is an overabundance of detailed posters and hand hygiene reminders throughout the hospital. Participants state they and others have become inured to their presence and rarely stop to read them:

FGD1: “We have no time on our shifts to stand around reading posters and we’re yet to see any visitors stop and have a read”.

Participants suggest one universal poster with simple dialogue suitable for all language levels would be a more effective tool:

FGD2: “Just “Please wash your hands”, big sign. A lot of doors are mirrored. As you walk in, they have got the glass thing there. Cover the glass up, “Wash your hands as you walk in”. Plain and simple. At the moment we have got five/six ways to wash your hands, it’s just all too much. We need to change it”.

Participants suggest the “poster overload” currently experienced could be addressed by complementary promotion activities.

FGD2: “In the past when wards hung streamers and balloons next to sinks, this attracted attention and prompted more people to wash their hands”.

Theme 3: “The personal value of hand hygiene: Like he said, it’s second nature to us”.

The third theme is titled “The personal value of hand hygiene: Like he said, it’s second nature to us”.

This theme represents three relevant perceptions: participants’ personal hand hygiene practice, an increased mindfulness in their day to day lives, and, in turn, reflection about hand hygiene at work.

Participants feel the practice of hand hygiene inherited from their work environment has impacted on their personal habits. There is sense this comes from understanding the value of hand hygiene, and good practice is habitual.

FGD1: “It is not like I was a grub and I didn’t wash my hands beforehand, but I find that generally I guess it’s increased the importance of washing your hands. I think when you are working here, been here for a while, it becomes more, like he said, second-nature”.

Participants further reflect they are more mindful of hand hygiene practices in their day-to-day lives. There is a sense habits from work are pervasive as they go about their lives every day.

FG2: “I don’t even like to touch the toilet door. I use the paper I use to dry my hands with to open the door, always… [I]n elevators we will touch the buttons with our elbows instead of fingers because of germs”.

This increased mindfulness of hand hygiene prompted participants to suggest innovative ways of engineering better hand hygiene in their work environment. Suggestions include the installation of sensor taps and, for example, the construction of zig-zag toilet corridors to avoid contamination of freshly-washed hands:

FGD1: “You know how in shopping centres they have those zig-zag corridors to the bathroom so you don’t have to touch handles after you wash your hands, that’s a good idea”.

## 4. Discussion

This study provides evidence about hand hygiene-related attitudes amongst hospital cleaning staff, an under-researched professional group. The results of this study suggest hospital cleaning staff are aware of the importance of hand hygiene and perceive it to be a valuable activity. There is evidence that suggests hospital workers’ perceptions of hand hygiene as “valuable” is related to their adherence to optimal hand hygiene practices [10,11]. This study suggests the use of research findings about the value of hand hygiene in education for hospital cleaning staff is important: (1) in informing their perceptions of hand hygiene as a worthwhile activity; and (2) in motivating hand hygiene compliance. These are both fundamental aims of the NHHI [5].

The NHHI is, essentially, a “culture change” program [4,5]. This study identified support at all levels of the hospital hierarchy as important in creating a positive hand hygiene culture. The presence of role models, particularly senior staff who consistently display optimal hand hygiene practices, has a positive impact on hand hygiene compliance among hospital workers—a finding supported by other research [10,11,12,13]. Indeed, the NHHI highlights executive commitment and leadership as fundamental in improving hand hygiene practices in Australian hospitals [5].

In addition to leadership, this study found the support of peers is perceived by cleaning staff as important to achieving optimal hand hygiene practices. The NHHI also recognises peer groups as important in hand hygiene compliance [5]. Theories of behavioural psychology suggest that, in hospital worker populations, peer groups create pressure and increase capacity among themselves to engage in optimal hand hygiene practices [14,15,16].

This study reinforces the idea that involving hospital cleaning staff in the design and implementation of strategies to promote hand hygiene is critical to improving their hand hygiene practices. This study indicates cleaning staff have ideas for hand hygiene strategies which are relevant, feasible, informed and innovative, and they are keen to communicate these. Indeed, involving both clinical and non-clinical staff in the design, implementation and evaluation of hand hygiene programs for their workplaces, and thus providing them with a sense of program “ownership”, is a key goal of the NHHI [5,17].

Another aim of the NHHI is to develop a standardised approach to hand hygiene auditing [5]. This study found cleaning staff perceive hand hygiene audits to be a valuable feedback tool, an outcome reported in NHHI documents [5]. However, this study suggests cleaning staff do not consider audits to be a motivating factor for hand hygiene compliance. Despite this, there is evidence to suggest advertised or overt hand hygiene audits do increase hospital workers’ compliance with optimal hand hygiene practices, partly by creating pressure to conform to accepted hand hygiene norms [18,19]. Researchers suggest this effect may be “harnessed” to improve rates of hand hygiene compliance among hospital workers [18].

In this study, this effect of peer pressure in motivating hand hygiene compliance was harnessed through the provision of rewards and recognition for excellence in hand hygiene practices—for example, the distribution of “life saver” shirts. As has been found in other hospital worker populations [20,21], individual reward and recognition schemes are a motivating force for hand hygiene performance and could be used to drive practice improvements. Indeed, the NHHI promotes the use of rewards such as giveaways and spot-prizes as important in improving hand hygiene compliance [5]. There is limited evidence to suggest such interventions may positively influence hand hygiene-related behavioural change even more so than educational interventions [21,22]. However, the findings of this study suggest effective hand hygiene education remains a key factor in hand hygiene compliance.

This study identified several attitudes towards hand hygiene compliance, particularly in terms of hand hygiene education. One of the fundamental aims of the NHHI is to facilitate a national approach to hand hygiene, partly through the use of standardised education programs based on the Five Moments of Hand Hygiene message [23,24]. The NHHI advocates for the use of several different teaching tools in hand hygiene education [5]. However, this study identified using a wide variety of educational material has not helped to clarify hand hygiene practices and there is still confusion among cleaning staff regarding correct hand hygiene procedures. To reduce confusion and inconsistency, one set of streamlined materials—based on the widely-utilised Five Moments of Hand Hygiene philosophy and written in plain language—should be used.

The NHHI promotes posters as key hand hygiene education resources [5]. However, this study found visual reminders of the importance of hand hygiene compliance and hand hygiene practices (particularly posters) are viewed by hospital cleaning staff as largely ineffective. Not only do these resources have the same problems with inconsistency as do other hand hygiene education materials, this study also found hospital cleaners consider them overly abundant and too detailed. Hospital cleaning staff report they have become inured to the presence of such visual reminders. This is consistent with other Australian research [25,26]. Future hand hygiene campaigns need to avoid mixed messages, use simple, powerful wording and promote key activities to prevent hand hygiene “overload”.

As noted, the NHHI aims for a national standardised hand hygiene program [5]. However, this study suggests education about when specifically to perform hand hygiene needs to be customised for cleaning staff to focus on scenarios they are most likely to encounter in their work. This finding is supported by other Australian research [11]. There is research to suggest contextual issues impact significantly on hand hygiene compliance [27,28], and considering contextual issues in hand hygiene education will inevitably make this education more relevant and useful. This study suggests education should focus on reinforcing correct hand hygiene procedures—including information about when to perform hand hygiene and non-touch techniques should complement rather than replace correct hand hygiene practices [29]—in context, and this is supported by the NHHI [5].

The success of the NHHI has been confirmed in an 8-year longitudinal study examining outcomes [30]. Graysons’ findings affirm the success of the program, with Australia’s major public hospitals reporting declines in incidence of health-care associated *Staphylococcus aureus* bacteremia associated with the implementation of NHHI strategies. Amongst these findings, cleaning staff (included as “others” in Grayson’s study, along with administrative staff, students, personal care staff, ambulance officers and dental staff) exhibited a steady annual rise in hand hygiene compliance in the eight years since the NHHI was introduced [30]. Across all hospital staff, consistent monitoring and feedback of behaviour is recommended to maintain compliance with NHHI [30]. In addition, it is suggested that greater consideration be given to “personality profiling” when designing strategies for education and enforcement, to account for the personality differences between nursing, medical, allied health, and “other” support staff (including cleaning staff). Grayson et al. [31] found hospital support staff—an important group often neglected in infection prevention literature—require messaging based around a structured framework of rules and regulations, and clear, unambiguous directives that are highly protocol-based with no decision-making required. Our study provides further insight on how this successful programme can be further improved through complementary strategies which recognise the context of cleaning staff.

As the sample size in this study was small and relatively heterogeneous, and as all participants were from one hospital, the findings are limited in their generalisability. However, this study is valuable because it is the first to explore the attitudes of hospital cleaning staff in relation to hand hygiene in Australia and may have meaning for similar groups.

## 5. Conclusions

This exploratory qualitative study identified attitudes toward hand hygiene and the National Hand Hygiene Initiative (NHHI) amongst hospital cleaning staff. Despite their comparatively low rates of hand hygiene compliance, this group is keenly aware of the importance of hand hygiene. Findings indicate leadership and peer support are critical to creating a positive hand hygiene culture, and appropriate reward and recognition to those excelling in hand hygiene is a strong motivator. Barriers to hand hygiene in this group include the presence of multiple conflicting guidelines, an “overload” of hand hygiene information and a lack of customised education programs. Whilst the small cohort in this study means the findings are of limited generalisability, it is apparent hand-hygiene messages and training need to be more consistent and contextualised to achieve improvements in hand hygiene practices in hospital cleaning staff.

## Figures and Tables

**Table 1:** Demographics of focus group participants.

Variable		Count	%
Gender	Female	5	42%
	Male	7	58%
Age	<30 years	1	8%
	30–50 years	5	42%
	>50 years	6	50%
Time employed as a cleaner at this facility	0–3 years	3	25%
	3–10 years	4	33%
	>10 years	5	42%
How important is hand hygiene (scale 1–10)	Very important (score 10)	12	100%
How hard is hand hygiene (scale 1–10)	Very easy (score 10)	12	100%

## Appendix A. Focus Group Discussion Guide

Interview guide for Cleaning Staff Focus Groups

Introduction of researcher and project (5 min)

In this time introduce self as a QUT researcher, undertaking the study as part of VRES scholarship and Queensland Health initiative.

Explain the description of the study and what is hoped to be achieved during the interview.

Identifying values, attitudes and beliefs (50–60 min)

Can you tell me about the value you place on hand hygiene to yourself personally?

Probes: At home? family?

In your job/role, can you describe to me the value you place on hand hygiene?

Probes: professionalism? responsibility?

Can you describe to me how you feel about the practice of hand hygiene?

Probes: mechanical (frequency, length) + resources (soaps, antimicrobials)

Can you tell me about how hand hygiene fits into your daily work?

Probes: complicated? hurried?

Thinking about reminders around your workplace governing hand hygiene, can you tell how you feel about them?

Probes: posters? dispensers?

Tell me what you think about the guidelines surrounding hand hygiene in this hospital?

Probes: location? displayed?

How do you understand of the relationship between hand hygiene and infection control?

Probes:

Can you describe in detail to me the culture of hand hygiene in this hospital

Probes: supportive? Not important?

In relation to your employer, can tell me about how you much think this organisation values (or not) hand hygiene?

Probes: philosophy? support?

Thinking about education/professional development for hand hygiene, can you share your thoughts?

Probes: Five moments? helpful?

Closing and Final Questions (5 min)

Ask participant if there is anything they would like to add or if anything came up during the interview they would like to expand on.

Thank the participant for their time.
